# Evolution of Hydration Gel Phases and Microstructure in Alkali-Activated Binders with Varied Calcium Contents

**DOI:** 10.3390/ma19173623

**Published:** 2026-08-26

**Authors:** Qiang Zhang, Qing Wang, Zhaoyang Ding, Tianru Li, Mingyu Zhao

**Affiliations:** 1 School of Materials Science and Engineering, Shenyang Jianzhu University, Shenyang 110168, China; zhangqiangll0311@163.com (Q.Z.); dingzhaoyang@sjzu.edu.cn (Z.D.); litianru0114@126.com (T.L.); zmyzhaomingyu@sjzu.edu.cn (M.Z.); 2 School of Civil Engineering, Shenyang Jianzhu University, Shenyang 110168, China

**Keywords:** alkali-activated binders, CaO gradient, N-A-S-H gel, C-(A)-S-H gel, sol–gel synthesis, microstructural evolution

## Abstract

**Highlights:**

- Factor collinearity effects are decoupled by integrating grey relational and partial correlation analysis to identify real controlling factors for mechanical performance of alkali-activated binders.
- Sol–gel synthesized pure N-A-S-H and C-S-H reference gels were adopted to identify gel transition rules with different CaO content.
- Multiscale characterizations revealed how CaO dosage and Si/Al ratio synergistically control matrix densification and compressive strength.
- CaO content quantitatively regulated the relative proportion of N-A-S-H and C-(A)-S-H gels during geopolymerization.

**What are the main findings?**

- The mechanical performance of alkali-activated binders (AABs) is jointly controlled by CaO dosage and Si/Al molar ratio. CaO is the dominant factor dividing AABs into low-, medium- and high-calcium zones with distinct strength ranges, while Si/Al ratio mainly tunes strength variation within low- and medium-calcium systems.
- Raising CaO content triggers the phase transition from N-A-S-H and weak zeolites to dense C-(A)-S-H gels together with stable Ca-rich chabazite, which boosts matrix compactness, thermal stability and compressive strength.
- FTIR Gaussian fitting and SEM-EDS confirm CaO reorganizes aluminosilicate networks: Ca facilitates octahedral Al turning into tetrahedral Al, modifies Si-O-T vibration peaks, accelerates gel polymerization and generates uniform, dense C-(A)-S-H microstructure in high-calcium samples.

**What are the implications of the main findings?**

- Grey relational and partial correlation analyses distinguish independent effect-driven and collinearity-driven correlations of oxide molar ratios for AABs compressive strength. Apparently high grey relational degree of the *n*(CaO)/*n*(SiO_2_ + Al_2_O_3_) ratio in the low-calcium AABs system originates from collinearity with the *n*(SiO_2_)/*n*(Al_2_O_3_) ratio rather than its intrinsic independent contribution to compressive strength.
- Tuning CaO dosage can directionally control gel species and crystalline phases, offering a feasible strategy to simultaneously improve the mechanical and thermal durability of low-carbon alkali-activated binders.

**Abstract:**

Alkali-activated binders (AABs) represent eco-friendly alternatives to ordinary Portland cement. Nevertheless, the synergistic influences of calcium dosage and aluminosilicate stoichiometry on phase assemblage, gel nanostructure, and mechanical properties have not been fully elucidated. Herein, AAB samples with graded CaO contents were fabricated, and the co-regulatory mechanisms of CaO dosage and Si/Al molar ratio on compressive strength and microstructural evolution were systematically explored through compressive strength tests, XRD, TG-DTG, FTIR, and SEM-EDS. In addition, pure reference C-S-H and N-A-S-H gels were synthesized by using the sol–gel method for comparison with AAB pastes. The results reveal that CaO dosage acts as the primary parameter dictating gel phase transition and strength level, categorizing the prepared AABs into three distinct zones: low-calcium region (CaO < 10 wt.%), medium-calcium region (10–20 wt.%), and high-calcium region (CaO > 20 wt.%). Combined grey relational and partial correlation analyses clarify the collinearity-induced false correlations and reveal the stage-dependent independent effects of oxide molar ratios on AABs’ compressive strength. Low-calcium AAB matrices are dominated by N-A-S-H gel networks coexisting with abundant low-strength zeolite crystals, which deteriorate thermal stability and retard strength gain. Increasing CaO content triggers a progressive phase transformation from N-A-S-H gel to high-strength C-(A)-S-H gel. Abundant Ca-rich chabazite and C-S-H gel form in high-calcium systems, which fill internal pores and microcracks and greatly enhance matrix densification and thermal resistance. This work clarifies the multiscale regulatory mechanism of calcium species over gel polycondensation, crystalline phase development, and mechanical performance of AABs, offering fundamental theoretical guidance for the customized design and property optimization of high-strength alkali-activated binders.

## 1. Introduction

Ordinary Portland cement (OPC) dominates as the primary cementitious binder for global civil infrastructure. However, its limestone calcination process is extremely energy-intensive and releases massive CO_2_, accounting for 5–8% of global anthropogenic carbon emissions. The annual carbon output from cement production reaches 2.7–2.9 Gt, which has become a major bottleneck restricting the carbon neutrality goal of the construction industry [1]. Alkali-activated binders (AABs) have emerged as promising low-carbon alternative binders due to their capability of utilizing industrial solid wastes as raw materials and eliminating high-carbon calcination procedures [2]. Compared with OPC, AABs can reduce carbon emissions by approximately 80% and effectively alleviate dust pollution during production, exhibiting outstanding environmental and engineering application potential [3,4,5,6].

The formation of AABs relies on a typical dissolution-polycondensation mechanism, which is fundamentally different from the hydration reaction of OPC. Reactive aluminosilicate precursors dissolve in alkaline activators and further polymerize to form dense gel matrices with mechanical strength [7]. Classic theories, including Purdon’s alkali activation hypothesis [8], Malone’s zeolite phase formation theory [9], and Davidovits’ alkaline geopolymer theory [10], have systematically explained the basic reaction mechanism of AABs. Notably, the calcium content of raw materials dominates the gel phase type and macroscopic performance of AABs. Low-calcium precursors mainly form disordered N-A-S-H gel networks, while high-calcium slag-based systems generate layered C-(A)-S-H gels [11,12]. The two typical gel structures possess distinct differences in microstructure, mechanical properties, and thermal stability, resulting in significant performance heterogeneity of AABs with different calcium levels [13,14].

Existing studies have separately explored the properties of low-calcium N-A-S-H and high-calcium C-(A)-S-H AABs systems [15,16,17,18]. Nevertheless, current research still has prominent limitations and unsolved scientific problems. First, most previous studies adopt narrow CaO-range raw material systems; the dataset does not cover a broad GGBS dosage range from 0 to 100%. The lack of systematic calcium gradient research hinders the establishment of unified performance zoning criteria for the SiO_2_–Al_2_O_3_–CaO ternary system. Second, the synergistic regulation mechanism between CaO dosage and Si/Al molar ratio remains unclear. The coupled evolution law of gel nanostructure, phase assemblage, thermal stability, and compressive strength under interactive effects of calcium content and Si/Al ratio has not been quantitatively revealed. Third, existing investigations fail to capture the transitional microstructural evolution characteristics during the gradual transformation from N-A-S-H to C-(A)-S-H gel. Current studies can only characterize gels in specific regions using microscopic techniques such as NMR and SEM-EDS. There is a lack of a multi-scale characterization combination to construct a quantitative evidence chain for tracking the gel phase transition behavior of AABs.

To fill the above critical research gaps, this study systematically investigates the synergistic regulation effect of different CaO dosage and Si/Al molar ratio on the phase composition, microstructure, mechanical properties, and thermal stability of AABs. Pure N-A-S-H and C-S-H gel samples were synthesized for comparative analysis, and the variation law of relative content of two typical gels with calcium content was emphatically explored. Combined with compressive strength tests, XRD, TG-DTG, FTIR spectral deconvolution, and SEM-EDS multi-scale characterization, this study classified the low/medium/high calcium characteristic zones of the ternary system. The intrinsic synergistic mechanism of CaO content and Si/Al ratio governing matrix densification and performance evolution was quantitatively elucidated. The key innovation is the integration of grey relational and partial correlation analyses to remove collinearity-originated false correlations and uncover stage-wise independent regulatory rules of oxide molar ratios for the strength of AABs in different calcium zones. This work aims to provide systematic theoretical guidance for the precise component design and performance optimization of high-strength low-carbon AABs and further promote the high-value recycling of industrial solid wastes and low-carbon development of the construction industry.

## 2. Materials and Methods

### 2.1. Materials

Metakaolin (MK) was purchased from Guangdong Fuhua New Material Company (Meizhou, China). The ground granulated blast-furnace slag (GGBS) was purchased from Anshan Iron & Steel Company (Anshan, China). The silica fume (SF) was supplied by the Anmei Micro Silicon Powder Industrial Company (Anshan, China). These raw materials were used to produce the AABs. The chemical composition of the raw materials is listed in Table 1.

Sodium hydroxide, aluminum sulfate, and calcium chloride were purchased from Hengxing Chemicals Manufacturing Company (Tianjin, China). The sodium silicate solution with a modulus of 3.0 and 33 wt.% solid content was obtained from Fubaojia Chemical Material Co., Ltd. (Shenyang, China).

### 2.2. Specimen Preparation

**Figure 1:**
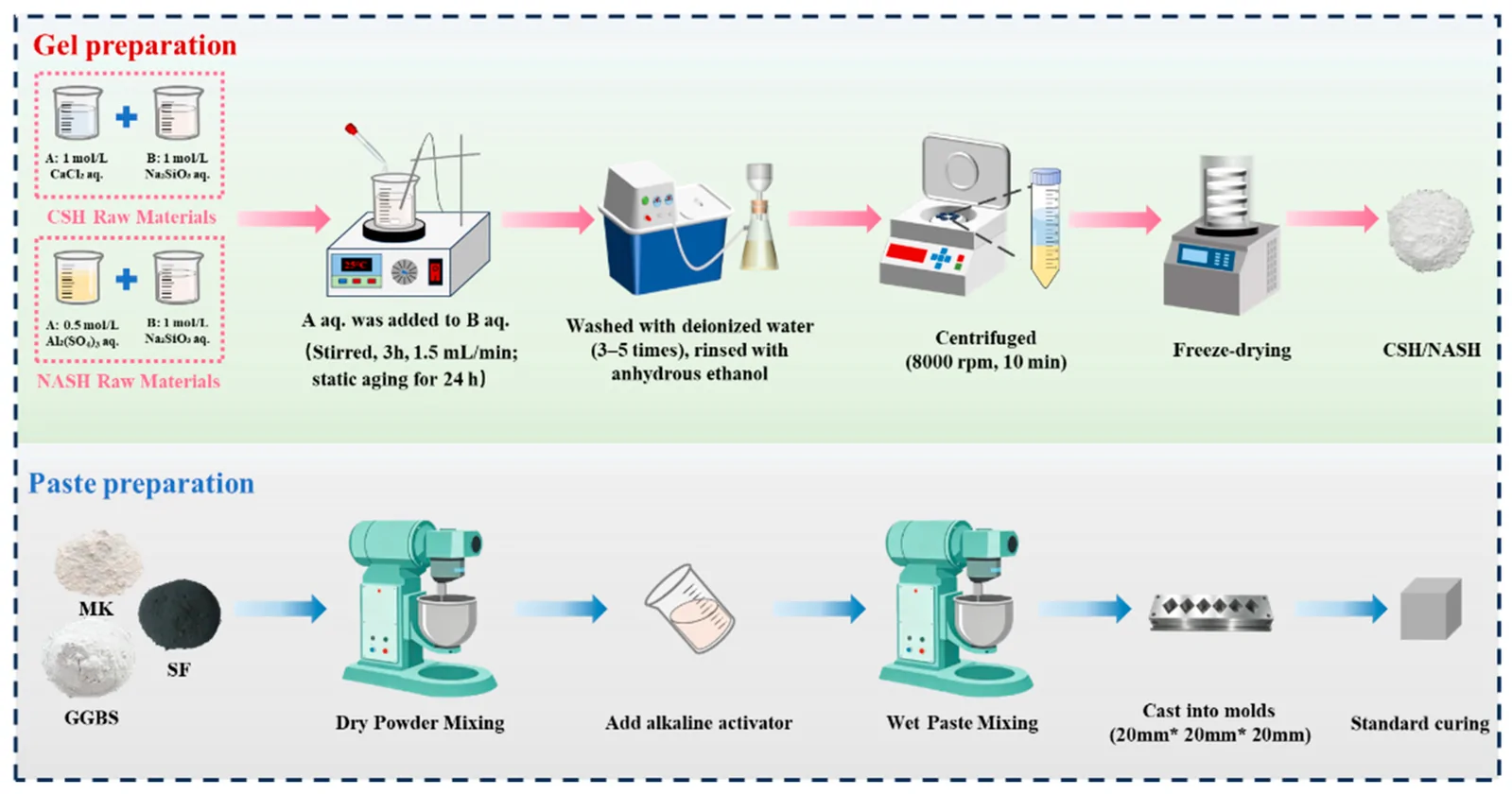
Preparation process of the gels and AAB pastes.

In this study, calcium silicate hydrate (C-S-H) and sodium aluminosilicate hydrate (N-A-S-H) gels were successfully synthesized via a conventional solgel method with consistent experimental procedures, except for the N-A-S-H gel, in which aluminum sources replace the calcium sources present in the C-S-H gel. AABs were prepared from slag, metakaolin, and silica fume under the activation of alkaline activators. Figure 1 illustrates the preparation process of the gels and AAB pastes.

For the synthesis of C-S-H gel, 1 mol sodium silicate solution was firstly prepared as the silicon precursor. Subsequently, 1 mol calcium chloride (CaCl_2_) solution was slowly dropped into the homogeneous sodium silicate solution under continuous magnetic stirring at room temperature to ensure sufficient and uniform reaction. After the dropwise addition was completed, the mixed suspension was continuously stirred at room temperature for 2 h and then statically aged for 24 h to fully complete the sol-gel reaction. Thereafter, the obtained suspension was centrifuged to separate the solid product. The collected solid precipitate was further freeze-dried at −55 °C under a vacuum pressure of 10 Pa for 48 h to remove residual moisture, yielding the final pure C-S-H gel product. The N-A-S-H gel was synthesized following the identical sol-gel route as the C-S-H gel, with the addition of 0.5 mol aluminum sulfate (Al_2_(SO_4_)_3_) as the aluminum source and 1 mol sodium silicate solution as the silicon precursor. The details of the gels are given in Table 2. The phase composition and purity of the synthesized C-S-H and N-A-S-H gels were characterized by X-ray diffraction (XRD). Both samples presented typical broad and diffuse amorphous diffraction humps in the characteristic diffraction range, which is a representative structural feature of gel materials. No discrete sharp diffraction peaks were observed, indicating the absence of unreacted raw materials and confirming the full reaction of precursors and high phase purity of the product.

**Figure 2:**
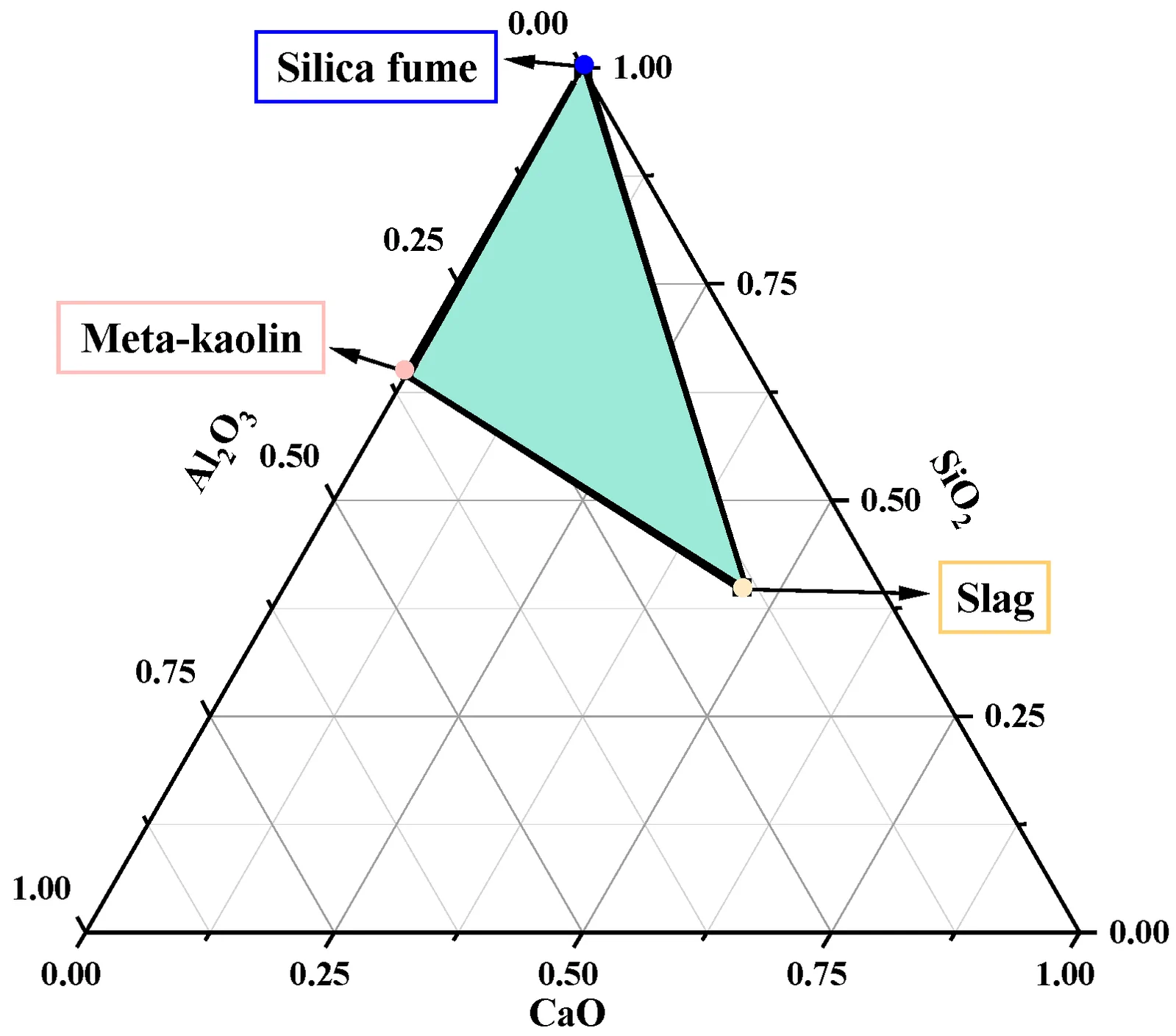
CaO-Al_2_O_3_-SiO_2_ ternary phase diagram.

The main oxide components dominating the reaction of AAB pastes are derived from SiO_2_, Al_2_O_3_, and CaO in raw materials. Based on the oxide composition of raw materials listed in Table 1, 62 groups of AABs with diverse oxide ratios can be fabricated by adjusting the dosages of the selected raw materials, covering the shaded region (green block) in the CaO-Al_2_O_3_-SiO_2_ ternary phase diagram (Figure 2). Various oxide ratio points within this shaded region can be achieved by regulating the contents of slag, metakaolin, and silica fume. The detailed powder mixing proportions are given in Table S1 of the Supplementary Materials. For the AABs, the alkali-activator was prepared with a water-glass modulus of 1.4. The solid content dosage of water glass was 12 wt.% of the powder materials, and the water-to-binder ratio was controlled at 0.4 (including the water contained in the water glass).

### 2.3. Methods

A thermogravimetric analysis (TG) study was performed on a STA449 F3 Jupiter simultaneous thermal analyzer (NETZSCH-Gerätebau GmbH, Selb, Germany) with a heating rate of 20 °C/min in air. A Powder X-ray diffraction (XRD) experiment was performed on an XRD-7000 X-ray diffractometer (Shimadzu Corporation, Kyoto, Japan) operated at 36 kV and 20 mA using Cu Ka radiation (λ = 0.15406 nm. The morphology of the samples was observed via scanning electron microscopy (SEM) using a Hitachi S-4800 field emission scanning electron microscope (Hitachi High-Technologies Corporation, Tokyo, Japan) with an accelerating voltage of 10 kV. The Fourier transform infrared spectroscopy (FT-IR) of the sample was measured using an Nicolet iS5 (Thermo Fisher Scientific, Madison, WI, USA). The chemical composition of the raw materials was analyzed using a S2-R X-ray fluorescence spectrometer (XRF) (Bruker AXS GmbH, Karlsruhe, Germany) operated at 144 eV energy resolution, and the power of the Pd target X-ray tube was 50 W.

### 2.4. Calculation Method for Grey Relational Degree (GRD)

(1) Determination of analysis sequences

The mother sequence *Y* = {*y*(*k*)| *k* = 1, 2, …, *n*} represents the 28-day compressive strength. The sub-sequences *Xi* = {*X_i_*(*k*)| *k* = 1, 2, …, *n*; *i* = 1, 2, …, *m*} correspond to the values of *n*(SiO_2_)/*n*(Al_2_O_3_) and *n*(CaO)/*n*(SiO_2_ + Al_2_O_3_).

(2) Dimensionless treatment of variables

Since each factor in the system possesses different dimensions, direct comparisons at the same level are not feasible. To obtain reliable relational degrees, the raw data are pre-processed for dimensionless transformation using Equation (1) prior to grey relational analysis.

(1) $$x_{i}(k)=\frac{X_{i}(k)}{X_{i}(l)},k=1,2,\ldots ,m$$

(3) Calculation of grey correlation coefficients

The grey correlation coefficients of *X_i_*(*k*) are calculated according to Equation (2).

(2) $$\xi_{i}\left(k\right)=\frac{\min_{i}\;\min_{k}\left|y\left(k\right)-\left.x_{i}\left(k\right)\right|+\rho \max_{i}\;\max_{k}\left|y\left(k\right)-\left.x_{i}\left(k\right)\right|\right.\right.}{\left|y\left(k\right)-\left.x_{i}\left(k\right)\right|+\rho \max_{i}\;\max_{k}\left|y\left(k\right)-\left.x_{i}\left(k\right)\right|\right.\right.}$$

The coefficient ρ is defined as the distinguishing coefficient, which is generally set to 0.5.

(4) Calculation of relational degrees

The correlation coefficients reflect the correlation degree between each sub-sequence and the mother sequence. The average value of a set of correlation coefficients is defined as the relational degree, and the formula for relational degree $r_{i}$ is shown in Equation (3).

(3) $$r_{i}=\frac{1}{n}\sum_{k=1}^{n}\xi_{i}\left(k\right),k=1,2,\ldots ,n$$

## 3. Results

### 3.1. Mechanical Properties

**Figure 3:**
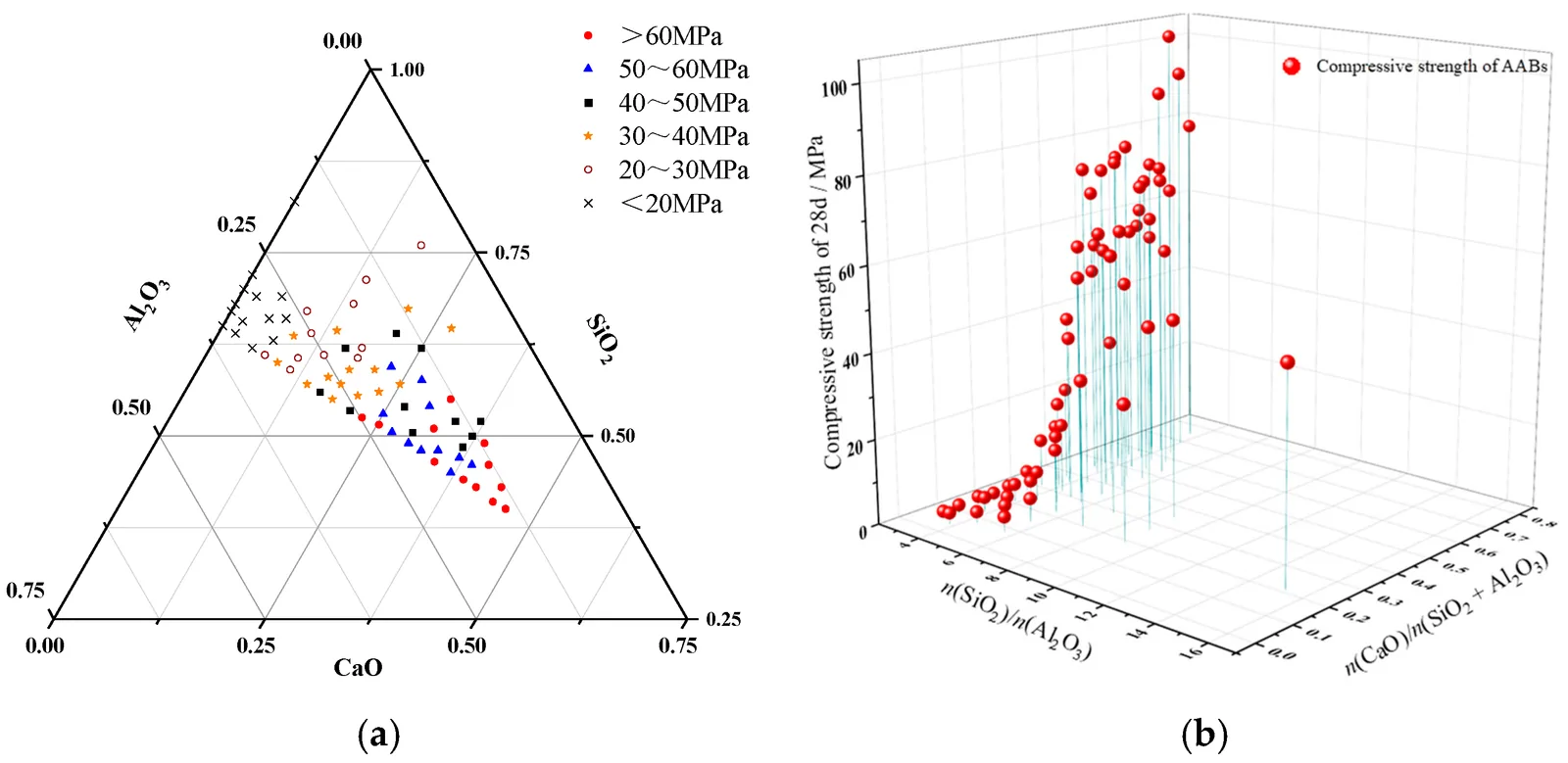
Compressive strength distribution of AAB pastes. (**a**) Strength distribution in ternary diagram; (**b**) 3D composition strength scatter plot.

Figure 3a presents the strength distribution region of AABs in the SiO_2_-Al_2_O_3_-CaO ternary system. Figure 3b illustrates the effects of different oxide molar ratios on 28-day compressive strength. The 28-day compressive strength of AABs in this system varies from 2 MPa to 102 MPa, showing a positive correlation with increasing CaO content. Specifically, AABs yield a compressive strength below 20 MPa when the CaO content is less than 10%. A moderate strength of 20–40 MPa is achieved at a CaO content of 10–20%. Further increasing the CaO content to above 20% results in a compressive strength exceeding 40 MPa. These findings demonstrate that CaO content is the predominant parameter governing the compressive strength of AAB pastes. On this basis, three characteristic zones, namely low-calcium, medium-calcium, and high-calcium regions, are defined for the AABs ternary system.

Based on the results in Figure 3, one group of experimental data was selected from the low-calcium, medium-calcium, and high-calcium regions. One representative AAB sample was selected from each region, and the corresponding oxide compositions of these samples are listed in Table 3. Microscopic characterizations were carried out on representative AABs from the three calcium content regimes to comparatively explore how calcium content affects reaction product evolution.

### 3.2. Microstructure Analysis

#### 3.2.1. XRD

**Figure 4:**
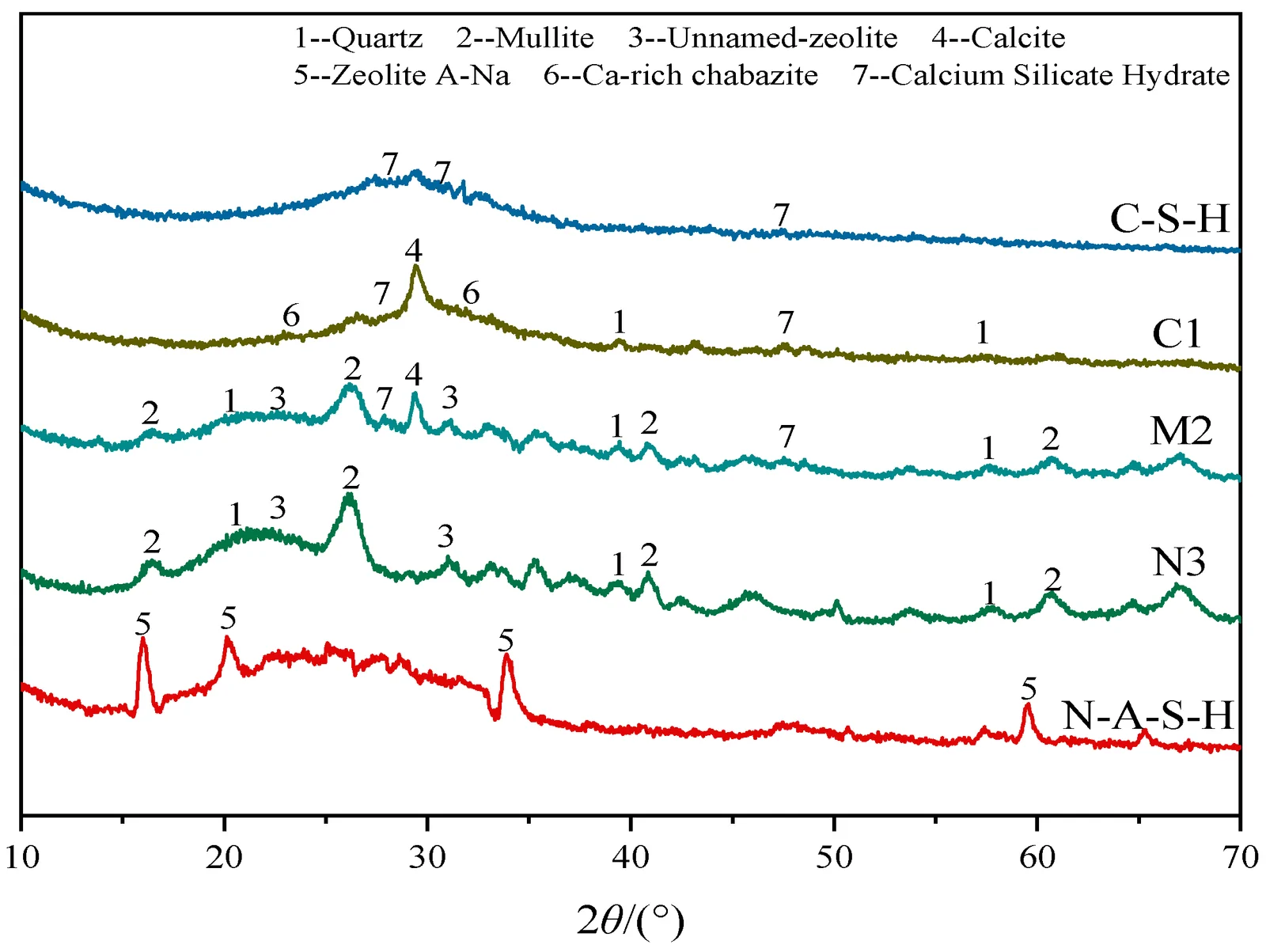
XRD patterns of gels and AABs.

Figure 4 presents the XRD patterns of the gels and AAB pastes. It can be seen from the diffraction pattern that the pattern of AABs is mainly amorphous. The broadening of the diffraction peaks in the amorphous state appears in the range from 20° to 35°, which is the characteristic X-ray diffraction of the AAB pastes [6,19]. Elevated calcium content induces a continuous shift of the characteristic amorphous scattering hump to larger 2*θ* angles. Such displacement originates from structural rearrangement of the aluminosilicate network and the formation of Ca-bearing amorphous C-(A)-S-H gel [20]. Besides the amorphous diffraction hump, the five samples display characteristic diffraction peaks corresponding to quartz, mullite, zeolite A, an unknown zeolite, Ca-rich chabazite, C-S-H gel, and calcium carbonate.

The highly cross-linked integral network of N-A-S-H gel features partial local ordering of precursors, which facilitates the crystallization of zeolite A in as-prepared N-A-S-H samples. However, crystalline zeolite phases can hardly crystallize within C-S-H gel, which is the main difference between the two pure gels. In addition to the broad diffuse hump characteristic of amorphous gel matrices, the XRD patterns of N3 paste mainly consist of unnamed zeolite, quartz, and mullite, which are very similar to N-A-S-H gels (Quartz and mullite represent unreactive mineral phases originating from the raw precursors). Both samples exhibit their amorphous humps at relatively large 2*θ* values. C1 paste is found to mainly contain hydrated calcium silicate, Ca-rich chabazite, quartz, and calcite without a zeolite phase, indicating that C1 paste was more similar to C-S-H gels (Quartz and calcite represent unreactive mineral phases originating from the raw precursors). The crystalline phase assemblage of M2 paste is more complex, with both zeolite and calcium silicate hydrate identified in the diffractogram. This suggests that the gel matrix of M2 likely contains a mixture of N-A-S-H and C-S-H gels.

#### 3.2.2. FTIR

**Figure 5:**
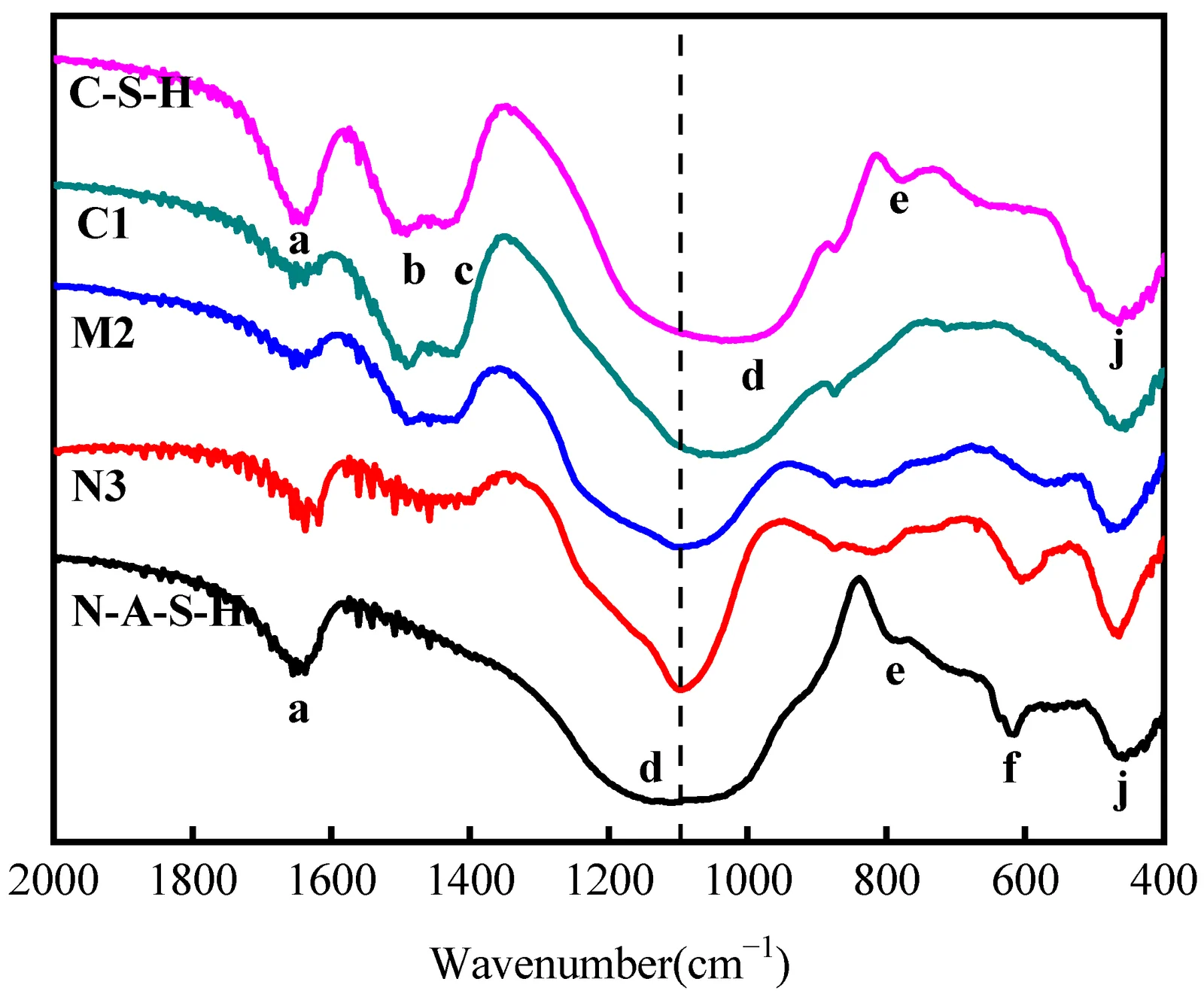
FTIR analysis of gels and AABs.

Figure 5 shows the FTIR spectra of C-S-H, N-A-S-H, and AAB pastes (N3, M2, and C1). Table 4 shows the characteristic absorbed peaks. The main characteristic peaks of each line (Si-O-T asymmetric stretching peak. T represents Si or Al.) are approximately 1112 cm^−1^~1091 cm^−1^ (band d). It shows that the main bonds of the pure gels and AAB pastes were all Si-O-T bonds. From bottom to top in Figure 5, each stacked infrared spectrum represents a sample with elevated CaO content, accompanied by a phase transformation from N-A-S-H through intermediate N3, M2, and C1 compositions to the final C-S-H gel. A progressive red shift of band d is observed as the CaO proportion rises. This indicates the three-dimensional network structure of the system is more complete [21]. The characteristic band centered at ~615 cm^−1^ (band f) arises from the ν_4_ asymmetric bending mode of non-framework octahedral [AlO_6_]. As Ca loading increases, this band weakens progressively, owing to the conversion of free octahedral Al into tetrahedral Al incorporated within the aluminosilicate network, which dominates the broad ν_3_ absorption at 980–1080 cm^−1^ [22]. C-S-H gel has two absorption bands at 1486 cm^−1^ and 1428 cm^−1^ (bands b and c). They are caused by non-symmetric vibrations and foreign bending vibrations of the CO (CO_3_^2−^) bond. But these bands cannot be found in the spectra of N-A-S-H gel. The bands arise primarily from surface carbonation via contact with ambient CO_2_ during curing or measurement as Ca^2+^ loading rises, instead of inherent peaks of the gel. Characteristic peaks centered around 750 cm^−1^ can be detected in the spectra of both N-A-S-H and C-S-H, originating from the ν_1_ symmetric stretching of Si–O and Al–O tetrahedral linkages. Compared with the band e of C-S-H, the corresponding band e for N-A-S-H shifts to higher wavenumbers, confirming that Al^3+^ substitutes Si^4+^ and integrates into the silicate tetrahedral framework [23].

#### 3.2.3. TG

**Figure 6:**
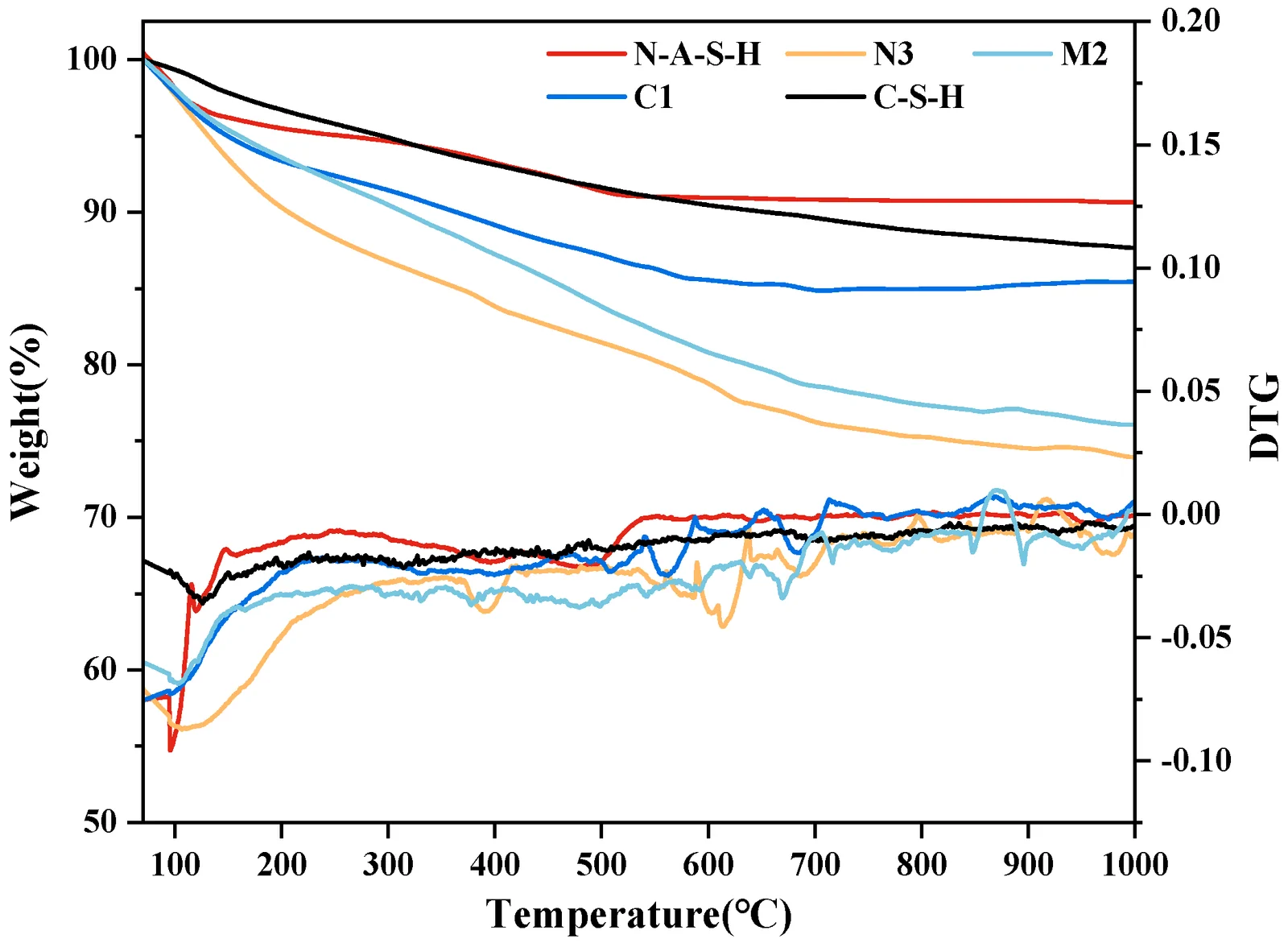
TG-DTG curves of gels and AABs.

Figure 6 presents the TG curves of synthesized gels and AAB pastes, which are used to evaluate the effects of calcium oxide content on hydration products and thermal stability. The mass losses of as-synthesized pure N-A-S-H and C-S-H gels are markedly lower than those of AAB pastes. This discrepancy mainly stems from the complex phase assemblages of AABs derived from industrial solid wastes after alkali-activated hydration. Major weight-loss peaks are detected between 60 and 230 °C, which correspond to the elimination of physically adsorbed water and the release of bound water from C-(A)-S-H gel [24]. Previous literature reported that the dehydration temperature of N-A-S-H gel is approximately 100 °C [25], consistent with the prominent DTG peak centered at 100 °C for pure N-A-S-H gel in Figure 6.

For AAB specimens, the weight loss at ~100 °C declines with increasing CaO content, revealing a reduction in the fraction of N-A-S-H gel within the matrix. A weak characteristic peak emerging at 130–160 °C is assigned to the dehydration of C-S-H gel. Sample C1 possesses a higher calcium dosage than Sample M2, facilitating the formation of larger amounts of C-S-H gel. Consequently, Sample C1 exhibits greater mass loss near 150 °C compared with M2.

The dominant weight-loss range of the second stage lies between 380 and 430 °C. Prominent DTG peaks are observed for pure N-A-S-H gel and N3 within this interval. M2 shows moderate peak intensity, whereas only negligible mass loss is detected for C1. The observed weight loss arises from the elimination of strongly bound cage water in zeolite crystalline frameworks. Compared with physically adsorbed pore water in amorphous gels, this zeolitic water requires an elevated temperature for desorption, consistent with the crystalline zeolite phases identified by XRD.

An endothermic peak assigned to decarbonation decomposition of carbonate phases emerges in the third stage at approximately 600–700 °C [26], which can be detected in all AAB pastes. Notably, the mass loss originating from carbonate decarbonation decreases with the increase of CaO dosage. This phenomenon can be explained by the fact that calcium ions preferentially participate in the formation of C-S-H gel, lowering the concentration of free Ca^2+^. Consequently, the samples suffer a weaker carbonation degree during preparation and curing. In summary, elevated calcium oxide content improves the high-temperature thermal stability of AABs.

## 4. Discussion

### 4.1. Effect of Calcium Contents on the Compressive Strength of AAB Paste

**Figure 7:**
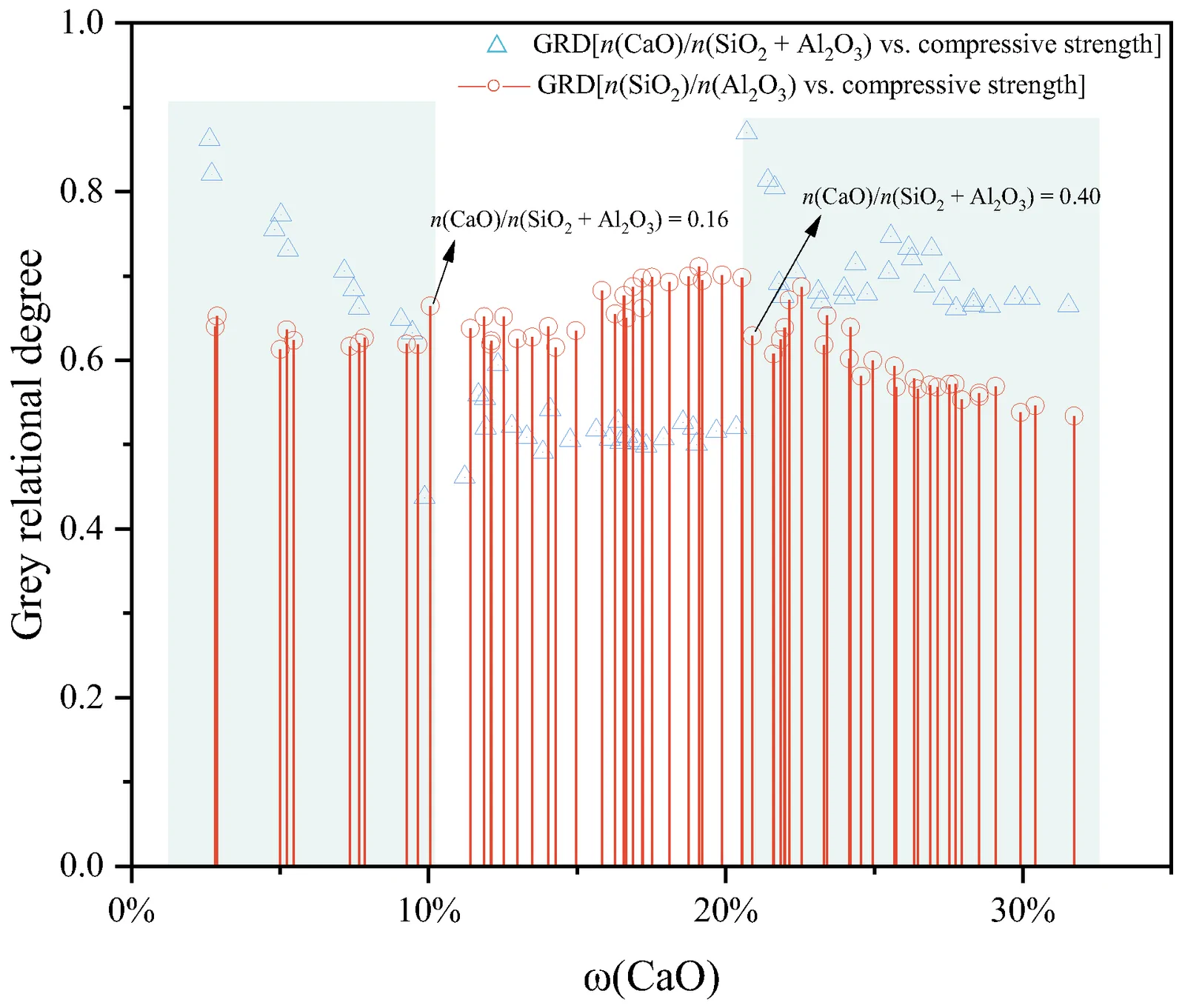
Grey relational degree of AABs under different calcium content.

Figure 7 presents the variation curves of grey relational degrees of two characteristic parameters, *n*(SiO_2_)/*n*(Al_2_O_3_) and *n*(CaO)/*n*(SiO_2_ + Al_2_O_3_), towards the 28 d compressive strength as a function of CaO mass fraction. At low CaO content (below 10%, corresponding to *n*(CaO)/*n*(SiO_2_ + Al_2_O_3_) = 0.16, the grey relational degree of *n*(CaO)/*n*(SiO_2_ + Al_2_O_3_) is generally higher than that of *n*(SiO_2_)/*n*(Al_2_O_3_). As the CaO mass fraction increases to the medium-calcium range of 10–20%, *n*(SiO_2_)/*n*(Al_2_O_3_) exhibits a higher grey relational degree, indicating that the dominant factor within the gel alters once the CaO mass fraction exceeds 10%. When the CaO mass fraction goes above 20%, the system enters the high-calcium region (*n*(CaO)/*n*(SiO_2_ + Al_2_O_3_) = 0.40), where the grey relational degree of *n*(CaO)/n(SiO_2_ + Al_2_O_3_) surpasses that of *n*(SiO_2_)/*n*(Al_2_O_3_) again. In this stage, calcium components regain independent effects, and calcium becomes the dominant factor of the system. It is also observed that both curves slightly decrease with further increasing CaO content, suggesting the occurrence of the calcium-saturation effect, whereby the overall relational degrees of the two parameters with compressive strength are weakened.

To explore the intrinsic correlation between the two independent variables, *n*(SiO_2_)/*n*(Al_2_O_3_) and *n*(CaO)/*n*(SiO_2_ + Al_2_O_3_), and the 28-day compressive strength, partial-correlation analysis was performed in this study. Within different calcium-content intervals, *n*(SiO_2_)/*n*(Al_2_O_3_) and *n*(CaO)/*n*(SiO_2_ + Al_2_O_3_) were sequentially set as control variables to eliminate coupling interference between the two factors, so as to evaluate the net correlation between each individual factor and the response indicator. The results are presented in Table 5.

Partial correlation results for samples in the low-calcium region show that a strong positive partial correlation exists between the *n*(SiO_2_)/*n*(Al_2_O_3_) ratio and compressive strength when *n*(CaO)/*n*(SiO_2_ + Al_2_O_3_) is controlled (R = 0.866, P = 0.003). When *n*(SiO_2_)/*n*(Al_2_O_3_) is controlled, *n*(CaO)/*n*(SiO_2_ + Al_2_O_3_) also exhibits a significant positive partial correlation with compressive strength (R = 0.722, P = 0.028). This indicates that both factors exert significant contributions to strength for the overall low-calcium dataset, while the independent driving effect of the *n*(SiO_2_)/*n*(Al_2_O_3_) ratio is more pronounced. These findings differ from the conclusion in Section 3.1 that *n*(CaO)/*n*(SiO_2_ + Al_2_O_3_) yields a higher grey relational degree. To interpret this discrepancy, the low-calcium region was further subdivided for comparative analysis into two sub-intervals (*n*(CaO)/*n*(SiO_2_ + Al_2_O_3_): 0.04–0.09 and 0.10–0.16), according to whether the grey-relational-coefficient difference ∆r exceeds 0.1 (see Table S1 in Supplementary Materials for detailed values). A value of ∆r > 0.1 denotes a notable gap between the grey relational degrees of the two factors, representing a considerable difference in the consistency of overall variation trends between the two factors and compressive strength across the dataset. The sub-interval results reveal that the partial-correlation coefficients between the *n*(SiO_2_)/*n*(Al_2_O_3_) ratio and compressive strength remain at extremely high levels (R > 0.94), whereas the partial correlations for *n*(CaO)/*n*(SiO_2_ + Al_2_O_3_) are all non-significant (P > 0.6). It can be concluded that the independent contribution of *n*(CaO)/*n*(SiO_2_ + Al_2_O_3_) to strength is unstable at the sub-sample scale within the low-calcium region. The statistical significance obtained for the full low-calcium dataset originates from enhanced statistical power caused by sample pooling. These statistical findings confirm that the strength of low-calcium AABs is predominantly governed by the *n*(SiO_2_)/*n*(Al_2_O_3_) ratio. Given the limited calcium availability in this region, the reaction is dominated by alkali-activated aluminosilicate polymerization to form N-A-S-H gel. The mechanical strength is controlled by the polymerization degree of Si-O-Al three-dimensional networks regulated by the *n*(SiO_2_)/*n*(Al_2_O_3_) ratio, while the direct effect of *n*(CaO)/*n*(SiO_2_ + Al_2_O_3_) is weak. The high grey relational degree observed for *n*(CaO)/*n*(SiO_2_ + Al_2_O_3_) arises from its collinearity with *n*(SiO_2_)/*n*(Al_2_O_3_), rather than its direct intrinsic contribution to strength. Accordingly, the *n*(SiO_2_)/*n*(Al_2_O_3_) ratio should be prioritized as the core tuning index for process optimization in the low-calcium regime.

Partial-correlation results for the medium-calcium region reveal an extremely significant positive partial correlation between the *n*(SiO_2_)/*n*(Al_2_O_3_) ratio and compressive strength when *n*(CaO)/*n*(SiO_2_ + Al_2_O_3_) is controlled (R = 0.687, P < 0.001). When the *n*(SiO_2_)/*n*(Al_2_O_3_) ratio is held constant, *n*(CaO)/*n*(SiO_2_ + Al_2_O_3_) also exhibits an extremely significant positive partial correlation with compressive strength (R = 0.692, P < 0.001), indicating that the two factors provide nearly equivalent independent contributions to strength. After subdividing the medium-calcium region into two sub-intervals according to calcium content, the partial correlation outcomes demonstrate the gradual evolution of the strength-governing mechanism. In the lower-medium-calcium sub-interval, a very strong positive partial correlation is observed between the *n*(SiO_2_)/*n*(Al_2_O_3_) ratio and strength under controlled *n*(CaO)/*n*(SiO_2_ + Al_2_O_3_) (R = 0.941, P = 0.002), whereas *n*(CaO)/*n*(SiO_2_ + Al_2_O_3_) shows no significant correlation when the *n*(SiO_2_)/*n*(Al_2_O_3_) ratio is controlled (R = −0.072, P = 0.878). Strength is dominantly controlled by the single factor of *n*(SiO_2_)/*n*(Al_2_O_3_) in this sub-interval. For the higher-medium-calcium sub-interval, both the *n*(SiO_2_)/*n*(Al_2_O_3_) ratio (R = 0.648, P = 0.007) and *n*(CaO)/*n*(SiO_2_ + Al_2_O_3_) (R = 0.566, P = 0.022) yield significant positive correlations with strength, and the system transitions into a two-factor co-regulation regime.

As for samples in the high-calcium region. When *n*(CaO)/*n*(SiO_2_ + Al_2_O_3_) is held constant, the partial correlation coefficient between the *n*(SiO_2_)/*n*(Al_2_O_3_) ratio and compressive strength is 0.050 (P = 0.810), revealing no significant net correlation. This demonstrates that the *n*(SiO_2_)/*n*(Al_2_O_3_) ratio exerts no independent effect on strength under high-calcium conditions. By contrast, when the *n*(SiO_2_)/*n*(Al_2_O_3_) ratio is controlled, *n*(CaO)/*n*(SiO_2_ + Al_2_O_3_) exhibits a moderately significant positive partial correlation with compressive strength (R = 0.559, P = 0.003), confirming that calcium content acts as the key independent factor governing mechanical performance within the high-calcium regime. Reaction products in high-calcium systems are dominated by C-(A)-S-H gel. The high-calcium environment compresses the tuning range of the *n*(SiO_2_)/*n*(Al_2_O_3_) ratio, leading to a marginal contribution of the *n*(SiO_2_)/*n*(Al_2_O_3_) ratio to compressive strength. Comparison with the results of the medium-calcium region shows that the partial correlation coefficient of *n*(CaO)/*n*(SiO_2_ + Al_2_O_3_) gradually decreases with further increasing calcium content. This indicates that the linear regulatory effect of calcium on strength is weakened, and the system presents a calcium-saturation characteristic. Synthesizing the two-factor partial correlation results across different calcium-content regimes, the strength-control behavior of AABs exhibits a continuous evolutionary trend with increasing calcium content: single-factor dominance by the *n*(SiO_2_)/*n*(Al_2_O_3_) ratio → two-factor synergistic regulation of calcium-aluminosilicate components → calcium-saturation effect.

**Figure 8:**
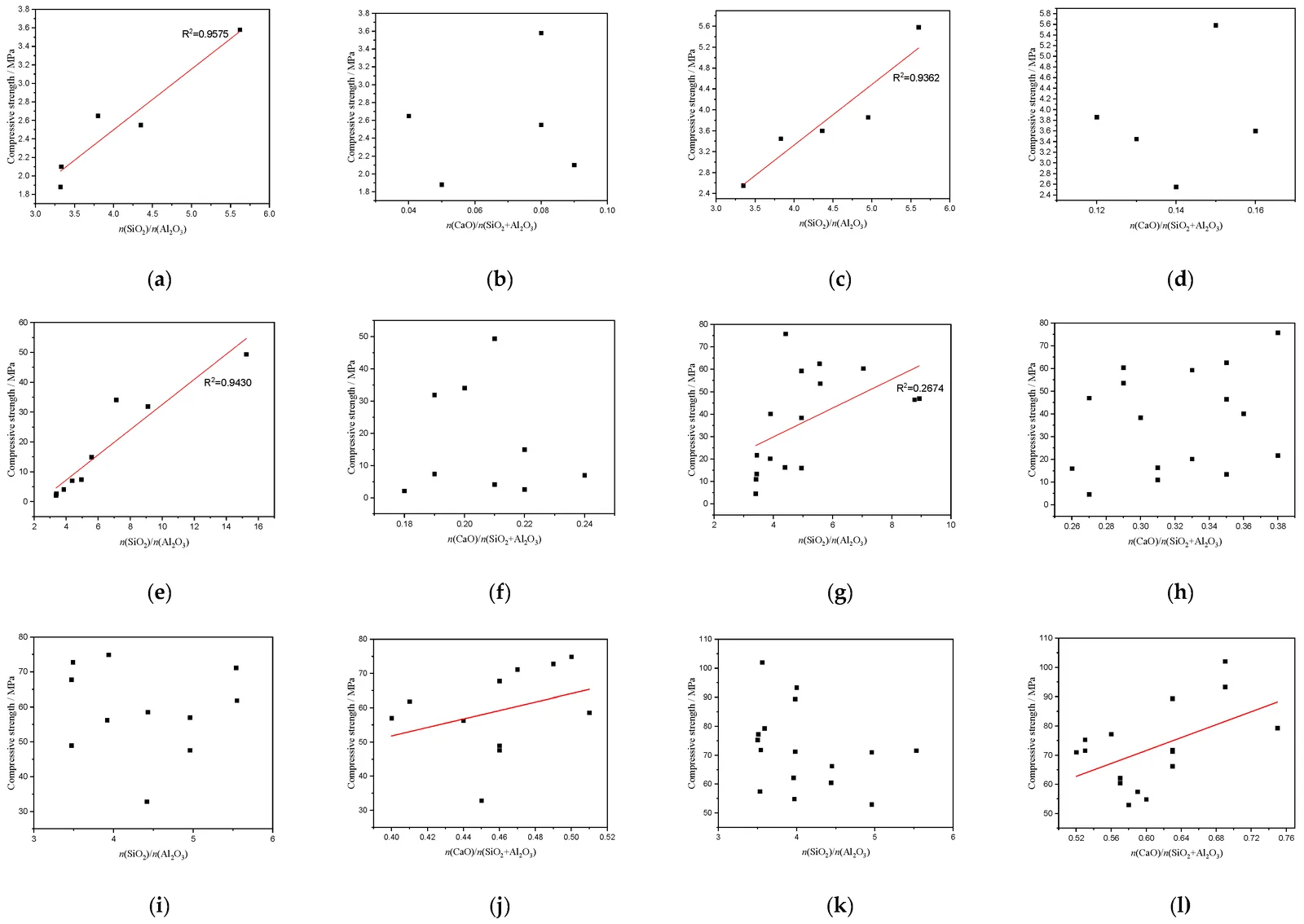
Linear fitting relationship. (**a**) low-Ca(0.04–0.08)-*n*(SiO_2_)/*n*(Al_2_O_3_); (**b**) low-Ca(0.04–0.08)-*n*(CaO)/*n*(SiO_2_ + Al_2_O_3_); (**c**) low-Ca(0.08–0.16)-*n*(SiO_2_)/*n*(Al_2_O_3_); (**d**) low-Ca(0.08–0.16)-*n*(CaO)/*n*(SiO_2_ + Al_2_O_3_); (**e**) Medium-Ca(0.16–0.24)-*n*(SiO_2_)/*n*(Al_2_O_3_); (**f**) Medium-Ca(0.16–0.24)-*n*(CaO)/*n*(SiO_2_ + Al_2_O_3_); (**g**) Medium-Ca(0.24–0.40)-*n*(SiO_2_)/*n*(Al_2_O_3_); (**h**) Medium-Ca(0.24–0.40)-*n*(CaO)/*n*(SiO_2_ + Al_2_O_3_); (**i**) High-Ca(0.40–52)-*n*(SiO_2_)/*n*(Al_2_O_3_); (**j**) High-Ca(0.40–52)-*n*(CaO)/*n*(SiO_2_ + Al_2_O_3_); (**k**) High-Ca(0.40–52)-*n*(SiO_2_)/*n*(Al_2_O_3_); (**l**) High-Ca(0.40–52)-*n*(CaO)/*n*(SiO_2_ + Al_2_O_3_).

Figure 8 presents the linear-fitting results of *n*(SiO_2_)/*n*(Al_2_O_3_) and *n*(CaO)/*n*(SiO_2_ + Al_2_O_3_) versus 28-day compressive strength for different calcium content regimes.

For the two sub-intervals within the low-calcium region, the coefficients of determination (R^2^) between *n*(SiO_2_)/*n*(Al_2_O_3_) and compressive strength reach 0.9575 and 0.9362, respectively, indicating an extremely strong positive linear correlation. By contrast, no obvious linear trend is observed between *n*(CaO)/*n*(SiO_2_ + Al_2_O_3_) and strength, demonstrating that strength development under low-calcium conditions is highly governed by the *n*(SiO_2_)/*n*(Al_2_O_3_) ratio. Upon entering the lower-medium-calcium sub-interval, the (R^2^) value for *n*(SiO_2_)/*n*(Al_2_O_3_) versus strength remains at a high level of 0.9430, and the single-factor-dominant feature of the *n*(SiO_2_)/*n*(Al_2_O_3_) ratio remains prominent. Nevertheless, within the higher-medium-calcium sub-interval, the (R^2^) of *n*(SiO_2_)/*n*(Al_2_O_3_) drops sharply to 0.2674. This reveals that the exclusive dominant effect of the *n*(SiO_2_)/*n*(Al_2_O_3_) ratio on strength is markedly weakened, and the system enters a two-factor synergistic-regulation stage. In the high-calcium regime, no linear correlation exists between *n*(SiO_2_)/*n*(Al_2_O_3_) and strength, whereas *n*(CaO)/*n*(SiO_2_ + Al_2_O_3_) shows a positive correlation trend with compressive strength. This reflects that the tuning range of the *n*(SiO_2_)/*n*(Al_2_O_3_) ratio is compressed under high-calcium environments, and calcium content becomes the dominant parameter controlling strength evolution. The fitting results suggest that *n*(CaO)/*n*(SiO_2_ + Al_2_O_3_) marks the onset of the dominant-factor transition interval, representing a critical compositional position where the system gradually shifts from *n*(SiO_2_)/*n*(Al_2_O_3_) ratio-dominated behavior toward calcium-involved synergistic effects. At this point, the calcium supply within the system reaches the threshold level for substantial formation of C-(A)-S-H gels.

### 4.2. Effect of Calcium Contents on the Phase Composition of AAB Pastes

As illustrated by the XRD spectra in Figure 4, chabazite is absent in low-CaO AAB pastes and continuously accumulates as the CaO dosage increases. Research results have shown that the compressive strength of zeolite-containing samples is lower than that of chabazite-containing samples [27,28], and when C-S-H is present in the sample, the compressive strength is significantly increased [29,30]. Research by JE Oh shows that AABs containing non-ABC-6 zeolites, such as analcime, zeolite X, and zeolite A, form less gel structure than AABs that contain chabazite and sodalite, and get low strength. Chabazite has a Si/Al molar ratio of 2, while Ca-rich chabazite exhibits a Si/Al ratio of 1. The two phases possess highly similar crystal structures regardless of their differing Si/Al ratios. Ca-rich chabazite promotes abundant gel generation, matching the function of chabazite documented in previous work. Simultaneous presence of Ca-rich chabazite and C-S-H in sample C1 explains its maximum mechanical strength.

FTIR results demonstrate that the spectra of C-S-H gel are more similar to Portland cement patterns [31,32], and FTIR spectra of N-A-S-H gel were more similar to fused silica [33], indicating that the former tend to be more like a layered structure of cement, but the latter tend to be a disordered internal three-dimensional network structure. AAB pastes display infrared absorption bands comparable to pure C-S-H and N-A-S-H gels, with the main difference being the morphology of the v_3_ Si–O–Si band near 1100 cm^−1^.

**Figure 9:**
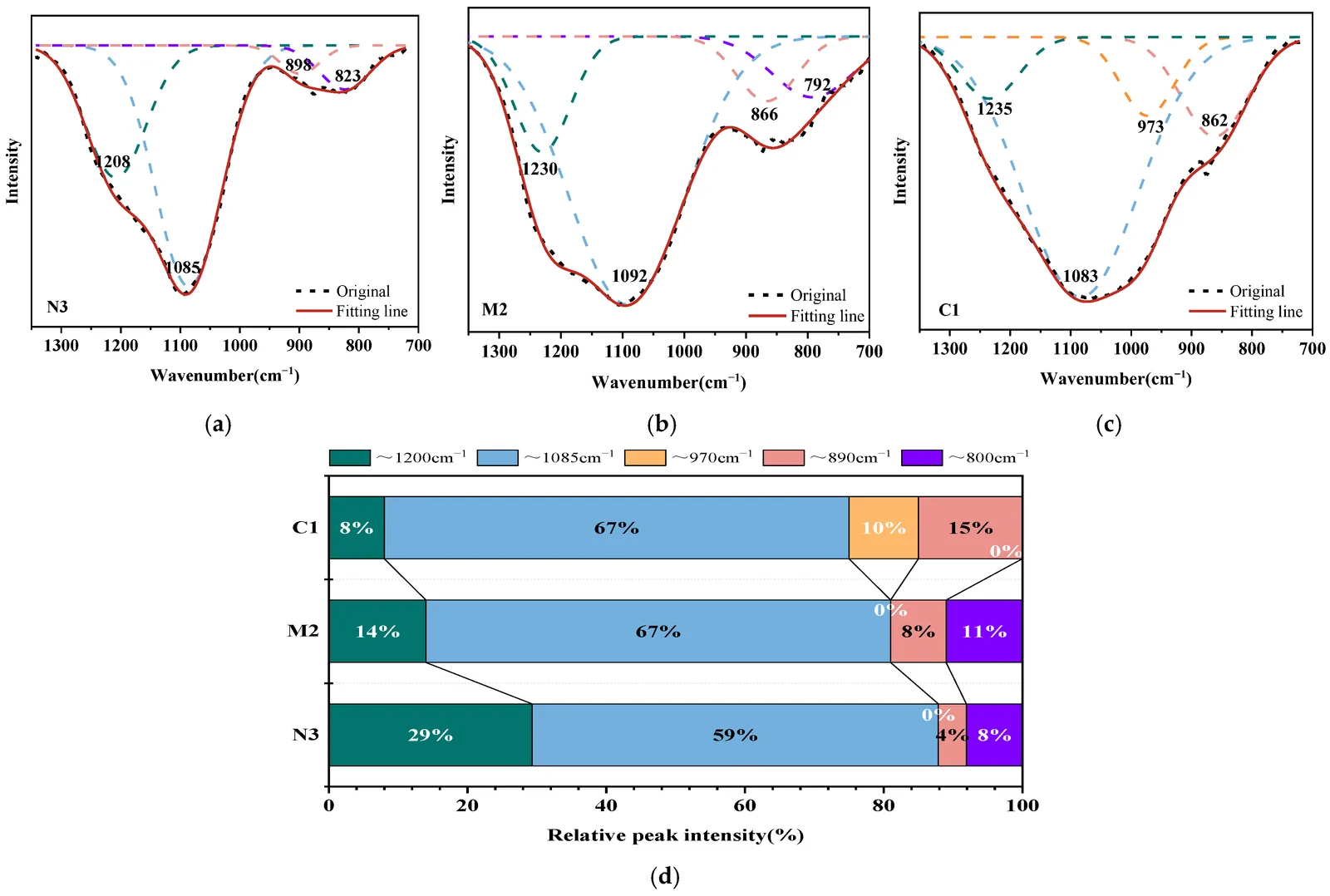
The deconvolution of the Si-O-T band of AABs. (**a**) Deconvolution of N3; (**b**) Deconvolution of M2; (**c**) Deconvolution of C1; (**d**) Peak-area fraction of deconvoluted sub-peaks.

To reveal the structural evolution of gel phases in AABs with varying CaO dosages, deconvolution fitting based on Gaussian functions was performed on FTIR spectra in the range of 700–1400 cm^−1^. The fitting results are presented in Figure 9. Remarkable variations in the FTIR profiles of AAB pastes are observed as the CaO content increases. Figure 9a–c correspond to the fitted spectra of Sample N3, M2, and C1 with gradually elevated calcium oxide dosage. All broad overlapping absorption bands were decomposed into individual characteristic sub-peaks via Gaussian deconvolution.

The dominant peak near 1085 cm^−1^ originates from the stretching vibration of Si–O–T bonds in unreacted metakaolin [34]. All samples were activated with alkaline activators of identical modulus, leading to comparable reactivity of the metakaolin raw material, which can be supported by the peak area fraction of residual metakaolin Si–O–T bands plotted in Figure 9d. A characteristic sub-peak centered at approximately 1200 cm^−1^ exists for all specimens, which is attributed to the framework vibration of silicon-rich aluminosilicate gel [35]; this sub-peak undergoes a continuous blue shift toward higher wavenumbers with increasing CaO addition, owing to the adsorption of divalent calcium cations onto the gel framework for charge balance [36]. Quantitative data in Figure 9d show that the peak area fraction of silicon-rich gel in Sample N3 reaches 29%, markedly higher than those of M2 and C1. This observation verifies that the silicon-rich N-A-S-H gel in AABs progressively transforms into C-(A)-S-H gel upon the incorporation of more calcium oxide.

A characteristic sub-peak at 973 cm^−1^ is observed for Sample C1, which is attributed to the residual raw slag phase [37]. The higher slag dosage in Sample C1 results in more unreacted slag components remaining after the alkali-activated reaction. The universal sub-peak near 890 cm^−1^ in all specimens corresponds to the stretching vibration of Si–O–Al bonds in silicate tetrahedral structures [38], verifying the successful incorporation of tetrahedral Al into silicate gel chains. With the increase in CaO content, this characteristic peak shifts continuously from 898 cm^−1^ to lower wavenumbers of 866 cm^−1^ and 862 cm^−1^. The peak at 862 cm^−1^ is assigned to the symmetric stretching of non-bridging Si–O bonds in low-polymerization-degree silicate tetrahedra [39]. This spectral evolution demonstrates that CaO incorporation facilitates the formation of C-(A)-S-H gel, which gradually replaces N-A-S-H gel and becomes the dominant gel phase in the AABs.

The absorption band near 800 cm^−1^ represents the symmetric stretching vibration of AlO_4_ tetrahedra [40]. For Sample N3, this peak appears at 823 cm^−1^, originating from the vibration of highly distorted AlO_4_ tetrahedra in unreacted metakaolin precursors, which is consistent with the low reaction degree of metakaolin concluded in Section 3.1. In Sample M2, the peak shifts to 792 cm^−1^. The increased calcium content accelerates the dissolution and reaction of Al species in metakaolin and promotes the formation of C-(A)-S-H gel. This process alleviates the structural distortion of AlO_4_ tetrahedra and reduces the vibration frequency of Al–O bonds, thereby causing a low-wavenumber shift of the characteristic peak [41]. According to the quantitative results in Figure 9d, the peak area fraction of Sample C1 at 800 cm^−1^ decreases to 0%. This indicates that C-S-H gel completely dominates the gel products of AABs under high-calcium conditions, with negligible N-A-S-H gel formed in the system.

### 4.3. Effect of Calcium Contents on the Microstructure of AAB Paste

**Figure 10:**
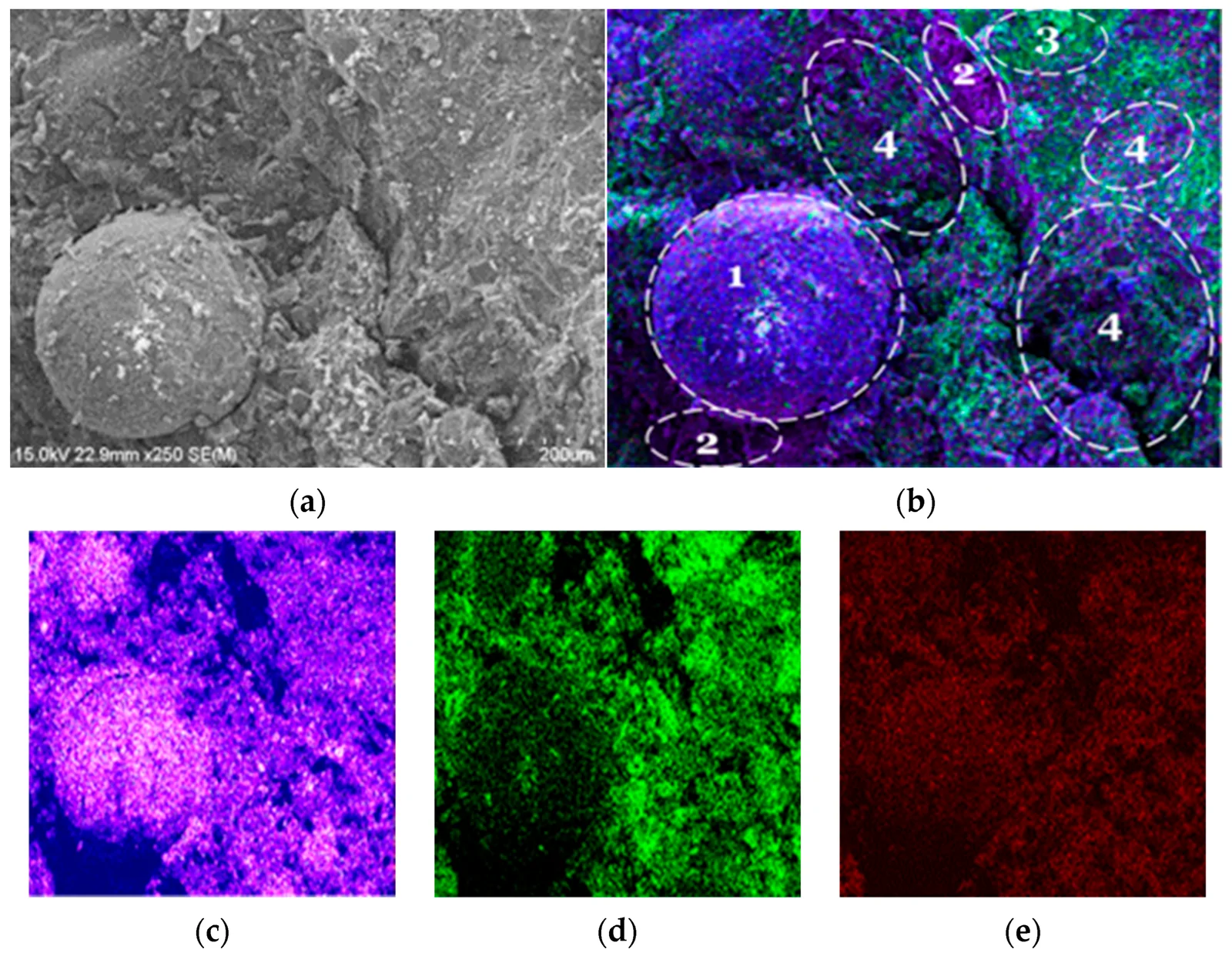

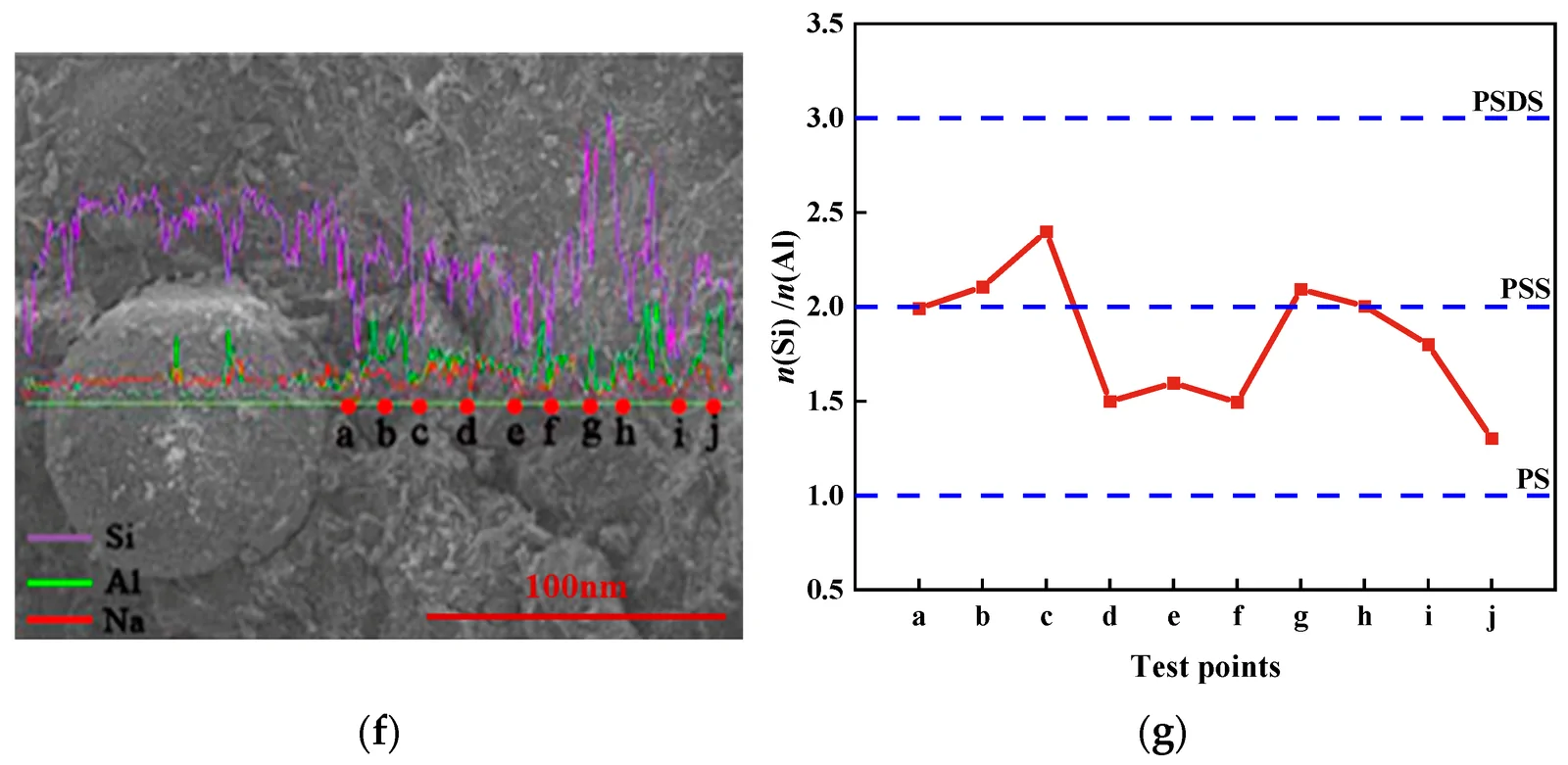
Element maps of N3 (**a**) SEM image; (**b**) Elemental surface scan; (**c**) Overlapping map of silicon; (**d**) Overlapping map of aluminum; (**e**) Overlapping map of sodium; (**f**) EDS elemental line scan; (**g**) Si: Al ratio of selected points.

Figure 10 presents the element maps of N3. The overlapping distribution map of Si (purple), Al (green), and Na (red) is displayed in Figure 10b, which can be divided into four distinct regions based on elemental contrast. Region 1 corresponds to residual silica fume particles. Regions 2 and 3 exhibit relatively homogeneous Si-dominated and Al-dominated distributions, respectively, originating from the dissolved and restructured silicon and aluminum species during alkali activation. According to previous research [42], such elemental distribution characteristics strongly suggest the predominant formation of N-A-S-H gel. Furthermore, the inhomogeneous and discontinuous elemental distribution demonstrates that the synthesized N-A-S-H gel possesses a heterogeneous microstructure during reaction, which reasonably explains the slow strength development of low-calcium AAB pastes. As reported by Somna [43], only N-A-S-H gels with uniformly distributed Si, Al, and Na elements can form a dense matrix and achieve superior mechanical strength.

Figure 10f illustrates the EDS line-scan results of the N3, and the corresponding Si/Al atomic ratios at ten testing points (a−j) along the scanning path are summarized in Figure 10g. EDS results confirm that the N3 contains no calcium species, and the major constituent elements (excluding oxygen) are Si, Al, and Na. According to Davidovits’s classical AABs theory [10], the three-dimensional network is constructed from three fundamental structural units: mono-silicate aluminum polymer (PS, *n*(Si)/*n*(Al) = 1), bi-silicate aluminum polymer (PSS, *n*(Si)/*n*(Al) = 2), and tri-silicate aluminum polymer (PSDS, *n*(Si)/*n*(Al) = 3). The measured Si/Al atomic ratios continuously fluctuate within the range of 1–3, with the overall values centered around 2. This indicates that the PSS unit serves as the dominant structural monomer in the calcium-free AABs. Meanwhile, coexisting PS and PSDS units endow the AABs network with heterogeneous molecular compositions and variable polymerization degrees.

**Figure 11:**
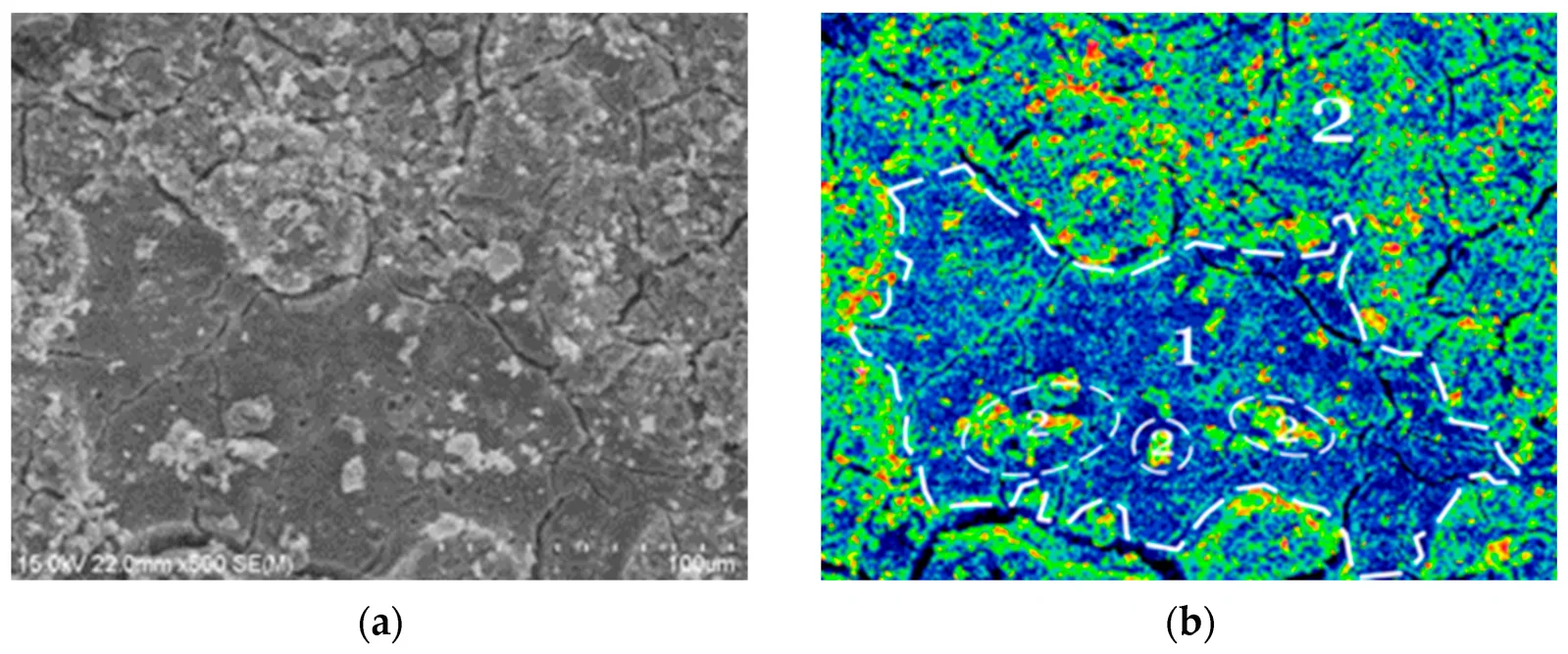

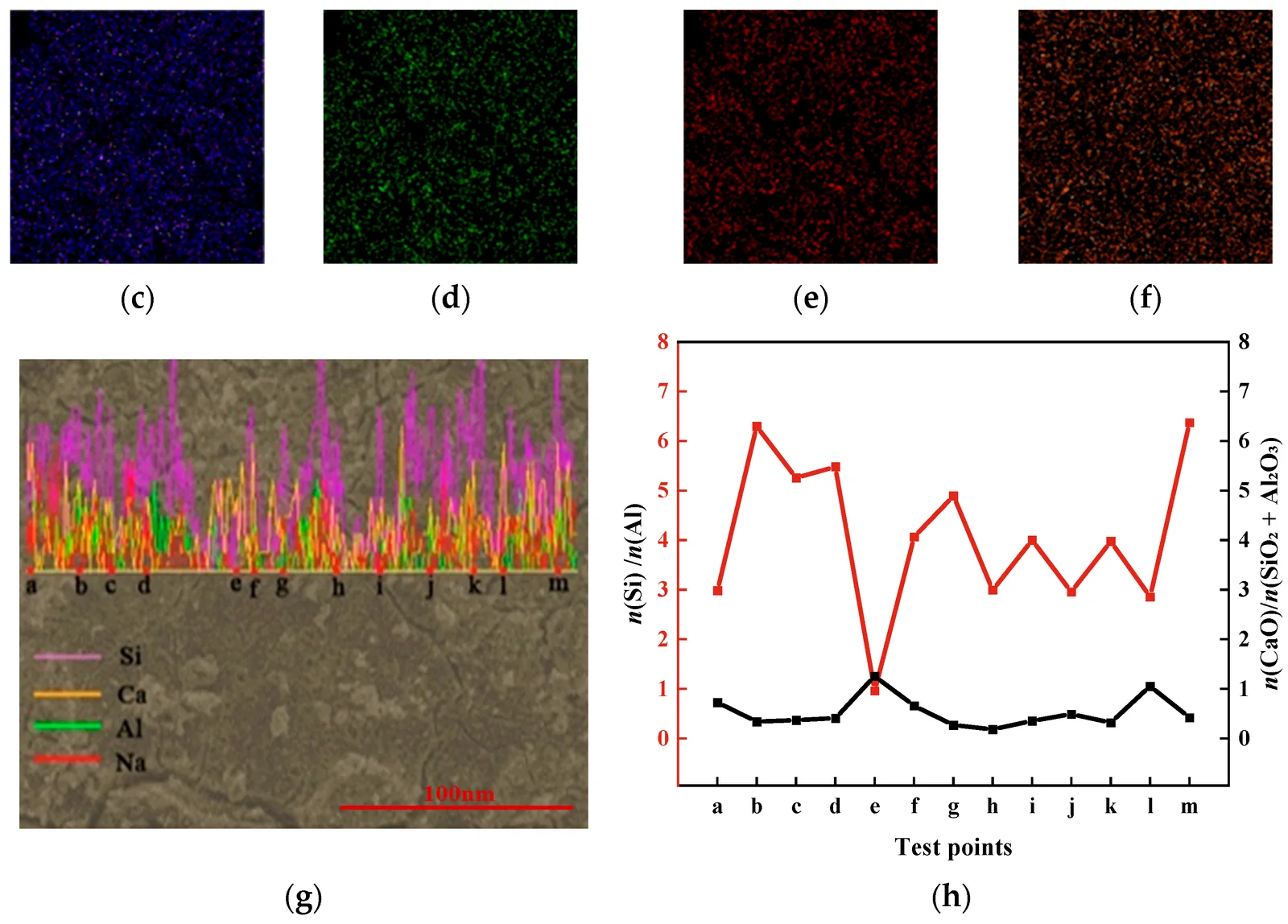
Element maps of C1 (**a**) SEM image; (**b**) Elemental surface scan; (**c**) Overlapping map of silicon; (**d**) Overlapping map of aluminum; (**e**) Overlapping map of sodium; (**f**) Overlapping map of calcium; (**g**) EDS elemental line scan; (**h**) Oxide molar ratio ratio of selected points, red line corresponds to *n*(Si)/*n*(Al), and the black line denotes *n*(CaO)/*n*(SiO_2_ + Al_2_O_3_).

Figure 11 presents the polished-surface EDS elemental scanning results of C1. The overlapping distribution map of Si (purple), Al (green), Na (red), and Ca (orange) is displayed in Figure 11b. Based on elemental distribution characteristics, the scanned area can be classified into two typical regions: a Si-rich region (Region 1) and a Ca-rich region (Region 2). Region 1 is dominated by Si, Al, and Na, demonstrating the formation of a three-dimensional N-A-S-H aluminosilicate network. In comparison, Region 2 exhibits a more complex elemental composition consisting of Si, Al, Ca, and Na with heterogeneous Ca distribution. High Ca concentration is concentrated at the core of residual slag particles, favoring the formation of C-S-H gels. At the slag surface, dissolved Al species participate in the reaction and further promote the generation of C-(A)-S-H gels. Consequently, mixed C-(A)-S-H gel phases are predominantly formed throughout region 2. Notably, the Ca-rich C-(A)-S-H gels are mainly distributed at the edges and cracks of the N-A-S-H matrix. This spatial distribution indicates that C-(A)-S-H phases are discontinuously dispersed along the boundaries of low-calcium N-A-S-H gels. The filling and cementing effect of high-density C-(A)-S-H effectively repairs micropores and cracks, thereby densifying the overall microstructure. This structural difference fundamentally explains the significantly higher compressive strength of C1 compared with N3 in Figure 9.

Figure 11g shows the EDS line-scan profiles of C1 paste, and the corresponding Si/Al atomic ratio and *n*(CaO)/*n*(SiO_2_ + Al_2_O_3_) molar ratio at thirteen detection points (a–m) are summarized in Figure 11h. EDS results verify that the main non-oxygen constituent elements of the C1 matrix are Si, Al, Ca, and Na. Low *n*(CaO)/*n*(SiO_2_ + Al_2_O_3_) values are observed at points b–d and g–h, implying limited calcium incorporation in these domains, which correspond to low-calcium C-(A)-S-H gels in Figure 11b. In contrast, point e exhibits a high *n*(CaO)/*n*(SiO_2_ + Al_2_O_3_) ratio with a balanced Si/Al ratio, yielding a Ca: Si: Al atomic ratio of 2:1:1 and confirming the formation of a high-calcium C-(A)-S-H gel. Point i presents the highest *n*(CaO)/*n*(SiO_2_ + Al_2_O_3_) ratio accompanied by an elevated Si/Al ratio, indicating relatively low Al content. This region corresponds to the slag core, where low-aluminum C-(A)-S-H gels are formed, consistent with the Ca-rich orange domain in Figure 11b.

Overall, the type and spatial distribution of hydration products in C1 are strongly dependent on the *n*(CaO)/*n*(SiO_2_ + Al_2_O_3_) molar ratio. It can be concluded that the *n*(CaO)/*n*(SiO_2_ + Al_2_O_3_) ratio serves as the dominant factor governing gel phase composition and ultimately determines the mechanical performance of high-calcium AAB pastes.

Although this work investigates the effects of CaO content on gel structures within AABs via macroscopic mechanical tests, microstructural characterizations and morphological analysis, several limitations remain to be addressed. All AAB specimens were cured under fixed ambient conditions, and the combined influences of curing temperature, humidity, and aging time on gel phase transition and matrix densification were not systematically investigated. In addition, the regulatory mechanism of calcium content was only analyzed from macroscopic and microscopic perspectives, without atomic-scale molecular dynamics simulations to further uncover the inherent evolution of Si–O–Al aluminosilicate networks. Further research will adopt variable curing systems and molecular dynamics simulations to supplement a multi-scale theoretical basis for the component design of high-performance low-carbon AABs.

## 5. Conclusions

The present investigation systematically elaborates the dependence of mechanical properties on calcium oxide dosage for AABs and elucidates the composition and structural transformation of gel assemblages in the matrix. The main conclusions are as follows:

1. The compressive strength controlling mechanism of AABs evolves continuously with CaO content: strength is predominantly governed by the *n*(SiO_2_)/*n*(Al_2_O_3_) ratio in the low-calcium region, shifts to two-factor synergistic regulation by *n*(SiO_2_)/*n*(Al_2_O_3_) and *n*(CaO)/*n*(SiO_2_ + Al_2_O_3_) ratio in the medium-calcium region, and is controlled mainly by calcium loading in the high-calcium region, accompanied by a calcium-saturation effect. *n*(CaO)/*n*(SiO_2_ + Al_2_O_3_) < 0.25, *n*(SiO_2_)/*n*(Al_2_O_3_) maintains a high linear correlation with the 28-day compressive strength.
2. Increasing CaO content effectively regulates the phase composition, gel evolution, and thermal stability of AABs, achieving a continuous structural transition from N-A-S-H-dominated matrices with abundant low-strength zeolite phases to C-(A)-S-H-rich pastes containing high-stability Ca-rich chabazite. Such transformation reduces N-A-S-H gel content, enhances C-(A)-S-H formation, mitigates carbonation-induced mass loss, and consequently improves both the compactness and thermal stability of the AABs matrix, realizing a gradual enhancement in macroscopic mechanical strength.
3. FTIR spectral characterization and Gaussian deconvolution quantification demonstrate that increasing CaO dosage drives the continuous structural transformation of AAB gel networks from disordered three-dimensional N-A-S-H frameworks toward ordered, low-polymerization C-(A)-S-H dominated matrices; elevated calcium promotes the conversion of octahedral Al into tetrahedral Al incorporated within silicate chains, reduces distorted AlO_4_ units, induces systematic red/blue shifts of characteristic Si–O–T vibrations, and quantitatively decreases the fraction of silicon-rich N-A-S-H gel, ultimately reconstructing the gel structure toward cement-like C-S-H layered structures.
4. SEM morphological evolution and EDS characterizations reveal distinct reaction kinetics and gel microstructures among AAB specimens with different CaO contents. Low-calcium AABs form heterogeneous N-A-S-H networks dominated by PSS structural units with uneven element distribution, slow densification, and incomplete consolidation even at prolonged curing. High-calcium AABs exhibit drastically accelerated gelation and fully compacted microstructures filled with Ca-rich C-(A)-S-H gels.

## Figures and Tables

**Table 1:** Chemical composition of the raw materials [wt.%].

Composition	SiO_2_	Al_2_O_3_	CaO	MgO	K_2_O	Fe_2_O_3_	Na_2_O
MK	62.09	34.33	--	1.46	0.69	--	--
GGBS	36.77	13.91	34.24	11.68	0.47	1.98	0.5
SF	88.91	0.44	0.68	3.25	2.29	0.44	1.89

**Table 2:** Details of the synthesized gels.

Sample	Ca/Si	Si/Al	Sodium Silicate Modulus
C-S-H	1	--	1.0
N-A-S-H	--	1	1.0

**Table 3:** Chemical oxide compositions of representative specimens.

Sample	*n*(CaO)/*n*(SiO_2_ + Al_2_O_3_)	*n*(SiO_2_)/*n*(Al_2_O_3_)
C1	0.385	4.2
M2	0.192	3.9
N3	0	3.7

**Table 4:** FTIR spectra of gels.

Band	C-S-H gel		N-A-S-H gel	
	Wavenumber (cm^−1^)	Chemical Bond	Wavenumber (cm^−1^)	Chemical Bond
a	1632	v_4_ OH	1628	v_3_ OH
b	1486	v_3_ CO(CO_3_^2−^)	--	--
c	1428	v_3_ CO(CO_3_^2−^)	--	--
d	1091	v_3_ Si-O-T	1112	v_3_ Si-O-T
e	775	v_1_ Si-O	787	v_1_ Al-O
f	--	--	615	v_4_ Al-O(AlO_6_)
g	467	v_2_ Si-O-Si	467	v_2_ Si-O-Si

**Table 5:** Partial correlation analysis results.

Region	Influencing Factors			
	*n*(SiO_2_)/*n*(Al_2_O_3_)		*n*(CaO)/*n*(SiO_2_ + Al_2_O_3_)	
	R	P	R	P
low-Ca (0.04–0.16)	0.866	0.003	0.722	0.028
low-Ca (0.04–0.08)	0.962	0.038	−0.375	0.625
low-Ca (0.08–0.16)	0.942	0.058	0.377	0.623
Medium-Ca (0.16–0.38)	0.687	0.000	0.692	0.000
Medium-Ca (0.16–0.24)	0.941	0.002	−0.072	0.878
Medium-Ca (0.24–0.40)	0.648	0.007	0.566	0.022
High-Ca (0.40–0.76)	−0.050	0.810	0.559	0.003
High-Ca (0.40–52)	0.069	0.849	0.331	0.351
High-Ca (0.52–0.76)	−0.114	0.686	0.437	0.103

## Data Availability

The original contributions presented in this study are included in the article/Supplementary Materials. Further inquiries can be directed to the corresponding author.

## Supplementary Materials

The following supporting information can be downloaded at: https://www.mdpi.com/article/10.3390/ma19173623/s1, Table S1: Powder composition, oxide molar ratio and grey relational degree of AABs.

## Abbreviations

The following abbreviations are used in this manuscript:

AABs	Alkali-activated binders
N-A-S-H	Sodium-aluminosilicate hydrate gel
C-S-H	Calcium-silicate-hydrate gel
C-(A)-S-H	Calcium-(alumino)-silicate-hydrate gel, when aluminum partially enters the C-S-H structure, the phase is denoted as C-(A)-S-H
