# Supplementary material for: Evolution of Hydration Gel Phases and Microstructure in Alkali-Activated Binders with Varied Calcium Contents
Source: Materials (Basel). 2026 Aug 26;19(17):3623. doi: 10.3390/ma19173623 (PMC13566892; doi:10.3390/ma19173623)
Supplement: Supplementary file 1 [file materials-19-03623-s001.zip › materials-4500364-supplementary.pdf]

Table S1 presents the proportions of slag, metakaolin and silica fume for the 62 groups of AABs prepared in this paper. The oxide molar ratios are calculated based on the chemical compositions of raw materials. The grey relational degrees between each oxide molar ratio and the 28-day compressive strength are obtained using the calculation method described in Section 2.4.

Table S1. Powder composition, oxide molar ratio and grey relational degree of AABs

No.	GGBS /%	MK /%	SF /%	$n(SiO_2)/n(Al_2O_3)$	$n(CaO)/n(SiO_2+Al_2O_3)$	28d strength/ MPa	$r[n(SiO_2)/n(Al_2O_3)]$	$r[n(CaO)/n(SiO_2+Al_2O_3)]$	Δr
1	5	90	5	3.8	0.04	2.65	0.862	0.640	0.222
2	5	95	0	3.32	0.05	1.88	0.820	0.652	0.168
3	10	80	10	4.35	0.08	2.55	0.772	0.636	0.136
4	10	70	20	5.62	0.08	3.58	0.755	0.613	0.142
5	10	90	0	3.33	0.09	2.1	0.730	0.624	0.107
6	15	70	15	4.95	0.12	3.86	0.706	0.616	0.089
7	15	80	5	3.83	0.13	3.45	0.683	0.621	0.063
8	15	85	0	3.35	0.14	2.55	0.662	0.627	0.035
9	20	60	20	5.6	0.15	5.58	0.648	0.619	0.029
10	20	70	10	4.36	0.16	3.6	0.633	0.619	0.014
11	20	80	0	3.37	0.18	2.13	0.437	0.664	-0.227
12	25	60	15	4.95	0.19	7.41	0.461	0.638	-0.177
13	30	30	40	9.11	0.19	31.91	0.554	0.619	-0.065
14	30	40	30	7.12	0.2	34.09	0.595	0.652	-0.057
15	25	70	5	3.85	0.21	4.1	0.559	0.652	-0.093
16	40	0	60	15.25	0.21	49.39	0.542	0.615	-0.074
17	25	75	0	3.38	0.22	2.61	0.520	0.623	-0.103
18	30	50	20	5.59	0.22	14.93	0.522	0.625	-0.104
19	30	60	10	4.38	0.24	7.01	0.509	0.628	-0.119
20	35	50	15	4.95	0.26	15.98	0.505	0.635	-0.130
21	30	70	0	3.4	0.27	4.61	0.491	0.640	-0.149
22	45	15	40	8.93	0.27	47	0.503	0.650	-0.148
23	40	40	20	5.58	0.29	53.64	0.506	0.655	-0.149
24	45	25	30	7.04	0.29	60.38	0.502	0.662	-0.159
25	40	45	15	4.95	0.3	38.41	0.528	0.677	-0.149
26	35	65	0	3.41	0.31	11.05	0.517	0.683	-0.166
27	40	50	10	4.39	0.31	16.34	0.510	0.687	-0.177
28	40	55	5	3.89	0.33	20.2	0.505	0.697	-0.193
29	45	40	15	4.95	0.33	59.3	0.507	0.693	-0.186
30	40	60	0	3.43	0.35	13.41	0.498	0.699	-0.200
31	50	30	20	5.56	0.35	62.51	0.500	0.695	-0.194
32	60	0	40	8.77	0.35	46.43	0.520	0.698	-0.178
33	45	50	5	3.9	0.36	40.16	0.526	0.700	-0.173
34	45	55	0	3.44	0.38	21.7	0.520	0.711	-0.191

35	50	40	10	4.41	0.38	75.8	0.516	0.701	-0.185
36	55	30	15	4.96	0.4	57	0.870	0.629	0.241
37	60	20	20	5.55	0.41	61.88	0.805	0.625	0.180
38	55	40	5	3.92	0.44	56.28	0.813	0.607	0.206
39	60	30	10	4.42	0.45	32.84	0.705	0.687	0.018
40	50	40	0	3.47	0.46	48.94	0.676	0.672	0.004
41	55	45	0	3.47	0.46	67.8	0.691	0.639	0.052
42	65	20	15	4.96	0.46	47.6	0.669	0.653	0.016
43	70	10	20	5.54	0.47	71.16	0.674	0.639	0.035
44	60	40	0	3.49	0.49	72.78	0.681	0.618	0.063
45	65	30	5	3.94	0.5	74.88	0.685	0.602	0.083
46	70	20	10	4.43	0.51	58.58	0.678	0.600	0.078
47	75	10	15	4.96	0.52	70.99	0.704	0.593	0.111
48	65	35	0	3.5	0.53	75.28	0.715	0.581	0.134
49	80	0	20	5.53	0.53	71.58	0.733	0.578	0.155
50	70	30	0	3.51	0.56	77.24	0.747	0.568	0.179
51	80	10	10	4.44	0.57	60.45	0.732	0.568	0.164
52	75	20	5	3.96	0.57	62.16	0.720	0.566	0.154
53	85	0	15	4.96	0.58	52.96	0.702	0.572	0.131
54	75	25	0	3.53	0.59	57.44	0.688	0.570	0.118
55	80	15	5	3.97	0.6	54.86	0.673	0.571	0.102
56	90	0	10	4.45	0.63	66.23	0.664	0.569	0.095
57	85	10	5	3.98	0.63	89.35	0.671	0.561	0.110
58	85	10	5	3.98	0.63	71.25	0.666	0.557	0.108
59	80	20	0	3.54	0.63	71.8	0.661	0.553	0.108
60	95	0	5	4	0.69	93.29	0.673	0.546	0.127
61	90	10	0	3.56	0.69	102	0.674	0.538	0.136
62	100	0	0	3.59	0.75	79.31	0.665	0.534	0.131
